# Volar plating of dorsal PIPJ fracture-dislocations

**DOI:** 10.1186/1753-6561-9-S3-A47

**Published:** 2015-05-19

**Authors:** Winston Chew Yoon Chong

**Affiliations:** 1 Hand Surgery Associates, 329563 Singapore

## 

Fracture-dislocations of the proximal interphalangeal joint (PIPJ) remains a challenging injury to manage. For those that are unstable, a variety of surgical treatment have been reported, each with its pros and cons:

1. Open reduction and internal fixation, with

a. Interfragmentary screws from either the dorsal or volar approach,

b. Cerclage wiring

2. Hemi-hamate osteochondral grafting, using fixed with interfragmentary screws,

3. Volar-plate arthroplasty,

4. Hemi-arthroplasty replacement.

Volar plating of dorsal fracture-dislocation of the PIPJ with mini-T or hook plates ensures secure fixation and allows early mobilization with good results. This technique is also applicable when the volar fragment is comminuted.

The surgical technique of volar plating with mini-C arm guidance is as follows:

1. exposure through A3 pulley

2. mobilization of volar fragment attached to volar plate

3. dorsal blocking wire to reduce and hold joint in place

4. elevation of any depressed articular fragment and bone grafting

5. repositioning of volar fragment over the base of middle phalanx

6. preliminary fixation with K wire

7. plating with mini 1.2 or 1.3mm T plate, or hook plate(s)

8. removal of dorsal block wire if stable

**Figure 1:**
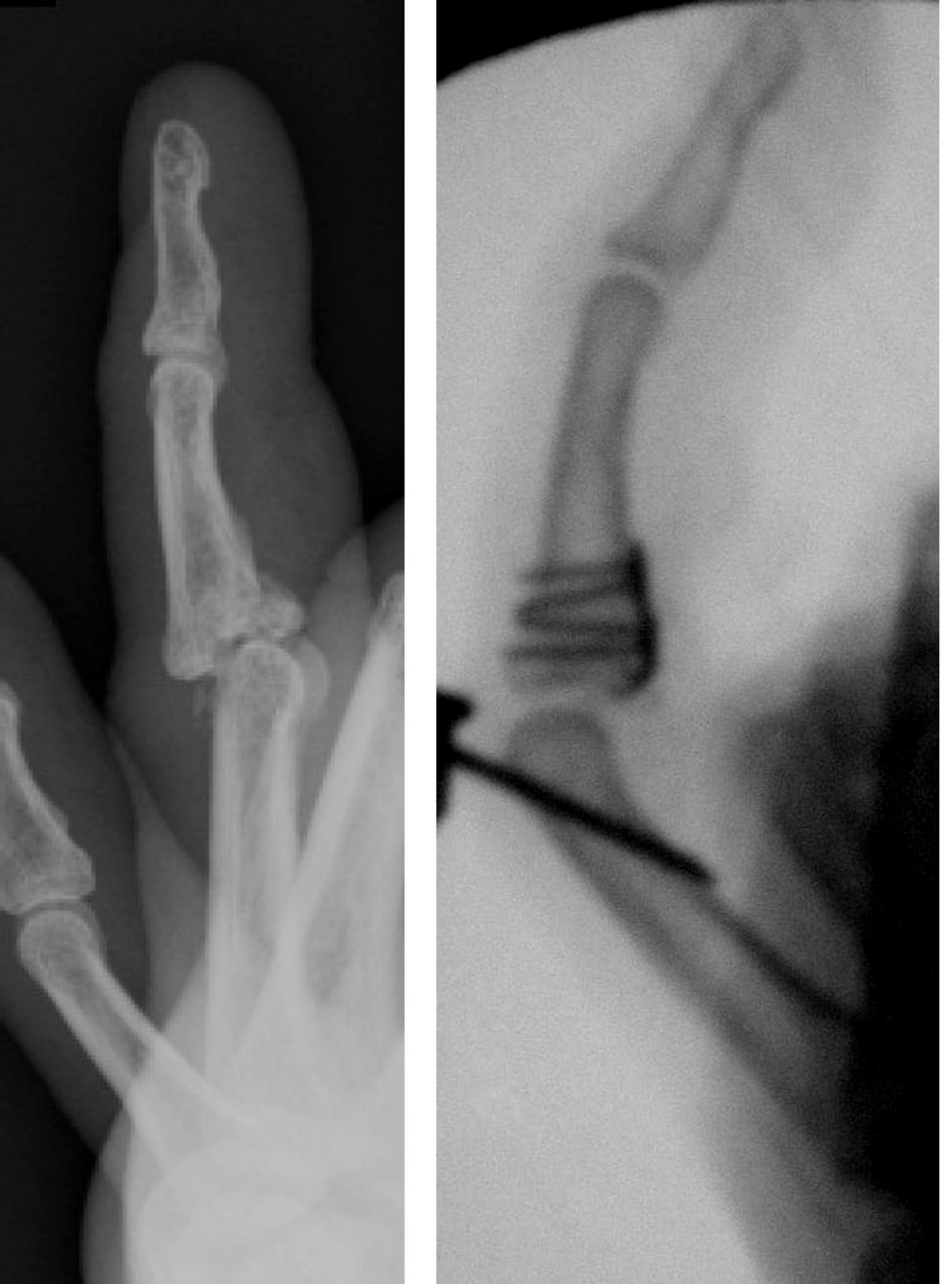


Early range of motion exercise regime is started. A resting gutter splint is applied to prevent flexion contracture of the PIPJ.
